# Genomic analysis reveals selection signatures of the Wannan Black pig during domestication and breeding

**DOI:** 10.5713/ajas.19.0289

**Published:** 2019-08-23

**Authors:** Wei Zhang, Min Yang, Yuanlang Wang, Xudong Wu, Xiaodong Zhang, Yueyun Ding, Zongjun Yin

**Affiliations:** 1 College of Animal Science and Technology, Anhui Agricultural University, Hefei 230036, China

**Keywords:** Wannan Black Pig, Selection Signature, Genome Variation, Porcine Industry

## Abstract

**Objective:**

The Wannan Black pig is a typical Chinese indigenous, disease-resistant pig breed with high fertility, and a crude-feed tolerance that has been bred by artificial selection in the south of Anhui province for a long time. However, genome variation, genetic relationships with other pig breeds, and domestication, remain poorly understood. Here, we focus on elucidating the genetic characteristics of the Wannan Black pig and identifying selection signatures during domestication and breeding.

**Methods:**

We identified the whole-genome variation in the Wannan Black pig and performed population admixture analyses to determine genetic relationships with other domesticated pig breeds and wild boars. Then, we identified the selection signatures between the Wannan Black pig and Asian wild boars in 100-kb windows sliding in 10 kb steps by using two approaches: the fixation index (F_ST_) and π ratios.

**Results:**

Resequencing the Wannan Black pig genome yielded 501.52 G of raw data. After calling single-nucleotide variants (SNVs) and insertions/deletions (InDels), we identified 21,316,754 SNVs and 5,067,206 InDels (2,898,582 inserts and 2,168,624 deletions). Additionally, we found genes associated with growth, immunity, and digestive functions.

**Conclusion:**

Our findings help in explaining the unique genetic and phenotypic characteristics of Wannan Black pigs, which in turn can be informative for future breeding programs of Wannan Black pigs.

## INTRODUCTION

Since their domestication some 9,000 years ago [1], approximately 300 breeds of pig (*Sus scrofa*) have been bred globally under natural and artificial selection [2] that have resulted in a range of adaptations and phenotypic features, which now distinguish those breeds from their wild counterparts. With the development of genetics and genomic technologies, more genes and genomes are being revealed [3,4], and as the cost of sequencing has declined, powerful tools are now available that can be readily used for studying the evolution of species and targeted selection to elucidate the involvement of natural processes and human technology in the evolutionary process and how both have shaped modern animal genomes to provide novel insights for further improving livestock.

A novel beneficial variant that has been under selection pressure usually shows a high population frequency and long-range linkage disequilibrium [5]. Geneticists have proposed a series of methods based on the decay of linkage disequilibrium and variation of allele frequency to detect genes under selection [6–8]. Numerous studies, based on chip or sequencing data, have been carried out to detect genome-wide selective signatures in humans [3,9] and in various agricultural species, including pigs [4,10,11], cattle [12], dogs [13], goats and sheep [14], chicken [8], and ducks [15] and have revealed a series of genes associated with hair development, skin pigmentation, coat color, body size, fertility, horn, environmental adaptation, adaptation to a starch-rich diet, and disease-resistance. Combined calculations of fixation index (F_ST_) [6] and π ratio [7] to detect selection signatures have been used in many studies [16].

The Wannan Black pig is a typical Chinese indigenous, disease-resistant breed with high fertility, and a crude-feed tolerance that has been bred in the south of Anhui province by artificial selection for a long time. Our previous study about retinol-binding protein 4 (*RBP4*) gene and cholesteryl ester-transfer protein (*CETP*) gene in Wannan black pig revealed that RBP4 was significantly associated with average back-fat thickness and meat color b* value, and identified a mutation in CETP, which had significant effects on the expression in liver and correlated positively with serum lipid and meat fat phenotypes [17,18]. Moreover, other genes and microRNAs related with immune traits and fertility have been identified [19,20]. However, these studies were performed only on a few genes and microRNAs. Further research to identify more genes that might assist us in elucidating the special characteristics of the Wannan Black pig breed, is required. Therefore, it is necessary to investigate selection signatures in Wannan Black pig to uncover its genetic characters.

Here, we focused on Wannan Black pig domestication and breeding using a resequencing dataset of 20 unrelated Wannan Black pigs and 28 other wild and domesticated pigs to find potential genomic evidence linking the domestication of Wannan Black pig with their breed characteristics on the basis of F_ST_ and π ratio. The findings herein will provide new insights to expand our understanding of the genetic base that determines the unique traits of the Wannan Black pig.

## MATERIALS AND METHODS

### Experimental animals and whole-genome sequencing

All experimental procedures were carried out in strict accordance with the protocols approved by the Anhui Agricultural University Animal Ethics Committee under permission No. AHAU20140215. Ear tissue was collected from 20 Wannan Black pigs (10 females and 10 males) from the Wannan Black pig conservation farm (Anhui, China) for high-throughput resequencing. DNA samples were extracted from all pigs using the Qiagen DNeasy Tissue kit (Qiagen, Dusseldorf, Germany), and the integrity and purity of the DNA were verified by agarose gel electrophoresis and A260/280 ratio. The genomic DNA was then processed with the Covaris system end-repair, A-tailing, ligation of pair-ended adapter, size-selection for sequencing and amplification. Finally, amplified fragments were sequenced on a HiSeqX platform using the protocols recommended by the manufacturer at Novogene (Beijing, China).

Additional resequencing data from 28 individuals were downloaded from NCBI (https://www.ncbi.nlm.nih.gov/), including 13 Asian wild boars, three European wild boars, and one of each of the following breeds: Yorkshire, Landrace, Bamaxiang, Rongchang, Meishan, Tibetan, Laiwu, Hetao, *Sus barbatus*, *Sus cebifrons*, *Sus verrucosus*, and *Phacochoerus africanus* (Supplementary Table S1).

### Reads alignment, variant calling, and annotation

To avoid low-quality reads, raw data were processed using the NGSQC Toolkit (v.2.30) [21]. Firstly, reads with an adapter sequence were deleted; next, reads containing more than 10% N bases were removed, and reads containing more than 50% low-quality bases (quality value ≤5) were discarded. Thus, only paired reads were preserved. The filtered pair-ended reads were aligned to the *Sus scrofa* reference genome (Suss11.1, GenBank assembly GCF_000003025.6) using BWA-MEM [22], and the bam file was sorted using Picard SortSam and improved using Picard MarkDuplicates (http://picard.sourceforge.net). Next, we used SAMtools mpileup [23] and GATK HaplotypeCaller algorithm [24] to call variants of the Wannan Black pig. Integrated variants from two algorithms were filtered using the tool “VariantFiltration” of GATK by “Qual ByDepth <20.0; ReadPosRankSum <−8.0; FisherStrand >10.0; Quality <MEANQUAL”, and then we recalibrated the bam file. Finally, the variants were called through GATK with “HaplotypeCaller” “GenotypeGVCFs” and “SelectVariants” parameter. To filter variants and avoid possible false positives, the tool “VariantFiltration” of GATK was used with the following options: “Depth <4.0; DP >1,000.0; RMS mapping quality <20.00” and “-cluster 2 -window 4” for single-nucleotide variants (SNVs); “QD <2.0; FS >200.0; ReadPos RankSum <−20.0” for insertions/deletions (InDels). After filtering, the variants were annotated with the ANNOVAR v2013-08-23 software [25]. This process of data filtering and variant calling was applied to sequencing data from Wannan pigs and other domesticated breeds and wild boars.

### Phylogenic construction, principle component analysis, and admixture analysis

**Figure 1:**
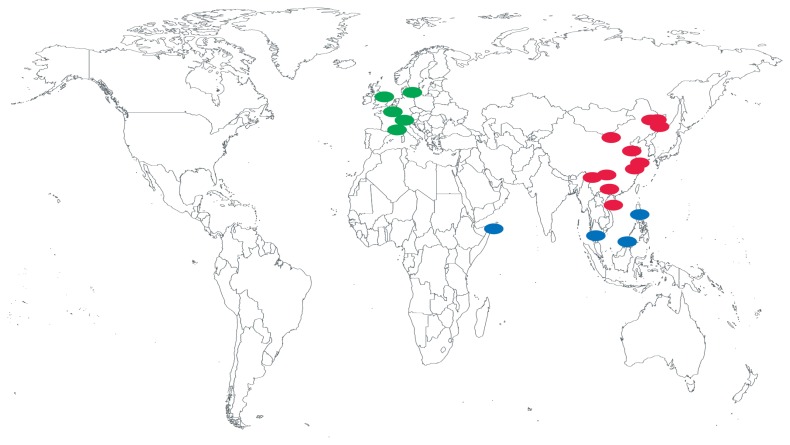
Geographic origin of the 19 analyzed pig breeds, including Europe and America (n = 5; green circles), Africa and Sus (n = 4; blue circle), Asia (n = 10; red circles).

To infer the population structure, we downloaded the sequencing data of 28 individuals from different geographical locations (Figure 1). To estimate the genetic and population structure of the pigs in our study, we filtered all autosome SNVs with a minor allele frequency (MAF) <0.05 and linkage disequilibrium (r^2^) <0.2 between studied pig populations, site missing rate <0.05, and quality value <30, and then we converted the filtered VCF file to PLINK input file formats (.map and .ped). First, we performed principle component analysis (PCA) with map and ped files using the GCTA software (v.1.25) using GCTA [26] “--make-grm” and “--grm tmp --pca 3” to generate .eigenval and .eigenvec files. Secondly, we performed a population admixture analysis using the ADMIXTURE software (v.1.3) [27] to infer the true number of genetic populations (clusters or K) among the pig breeds. Prior population information was ignored before testing and identifying distinct genetic populations and assigning individuals to populations. ADMIXTURE uses cross validation procedures to estimate the most preferable *K*-value, which is considered to be the one that exhibits a low cross-validation error, compared to other *K*-values and is considered to be the most probable number of inferred populations. Lastly, we performed a phylogenetic tree analysis by generating an identical-by-state distance matrix using the PLINK software v.1.90 (PLINK, RRID: SCR_001757) [28]. We then constructed neighbor-joining trees using SNPHhylo (v.2014 0701) [29]; trees were drawn using FigTree (v.1.4.0) [30].

### Detection of genome-wide selective sweeps

Regions under selection between the Wannan Black pig and the Asian wild boar were identified based on two different statistics, i.e. F_ST_ and π ratio. Prior to analysis, a series of quality-control procedures were implemented using VCFtools to achieve high-quality data by removing any SNVs with call rates <0.90 and with MAF <0.05 and by excluding individuals with a max missing count >4. Following quality control, missing genotypes were assigned using the BEAGLE software [31]. A 100-kb sliding window approach with 10-kb step-size was applied to calculate these statistics with PopGenome [32]. To define putative genomic regions under selection, first we defined the selection region of the two approaches based on 0.01 and 0.05 level. Then, the overlapped regions corresponding to 0.01 and 0.05 level of the two approaches were defined as the final selection signatures.

To explore the potential biological significance of genes within these sweep regions, gene ontology (GO) and Kyoto encyclopedia of genes and genomes (KEGG) analyses were carried out using Database for Annotation, Visualization and Integrated Discovery (DAVID, v.6.8) [33]. Benjamini-Hochberg false discovery rates [34] were used for correcting p values. Only terms with a p<0.05 were considered to be significant.

## RESULTS

### Genome resequencing and genetic variation

**Figure 2:**
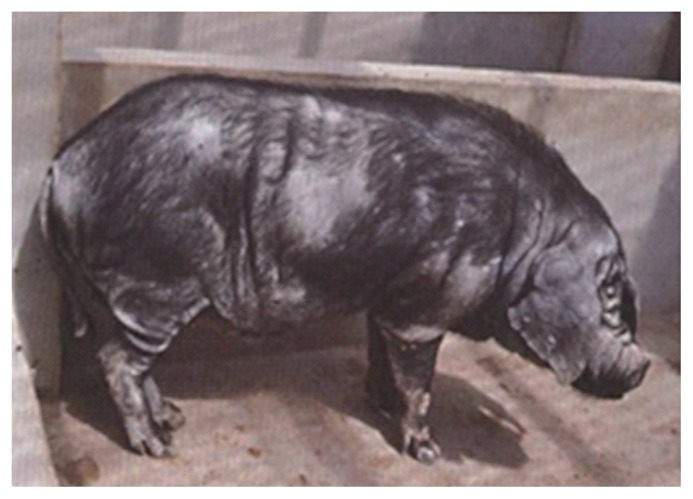
The Wannan Black pig.

Resequencing of the Wannan Black pig (n = 20, Figure 2) yielded 501.52 G of raw data. After mapping these to the *Sus scrofa* reference genome 11.1, an average of 84.74% (~83.44%–85.66%) of reads were mapped. The depth of the experimental population ranged from 7.10 to 11.38 folds with an average of 9.43. The depth of at least one or four of the experimental population averaged 98.02 and 89.31, respectively (Table 1). The data have been submitted to NCBI with accession number PRJNA524263.

After calling SNVs and InDels, we identified 21,316,754 SNVs and 5,067,206 InDels (2,898,582 inserts and 2,168,624 deletions). Among the SNVs, 3,896,353 were newly identified and were not included in the dbSNP database (ftp://ftp.ncbi.nih.gov/snp/organisms/pig9823/VCF/). Of all identified SNVs, intergenic variants were the most abundant (59.6%) (Table 2). Meanwhile, 144,815 were synonymous, 64,671 were non-synonymous, and 657 were stop-gain variants (Table 2). Additionally, we observed that the average ratio of transitions/transversions was 2.28. Of all identified InDels, most variants were in intergenic regions (59.5%). There were 13,766 in coding domains; meanwhile, 10,302 were frameshift variants, of which 6,447 were frameshift insertions and 3,855 were frameshift deletions (Table 2).

### Phylogenic construction, principle component analysis and admixture analyses

**Figure 3:**
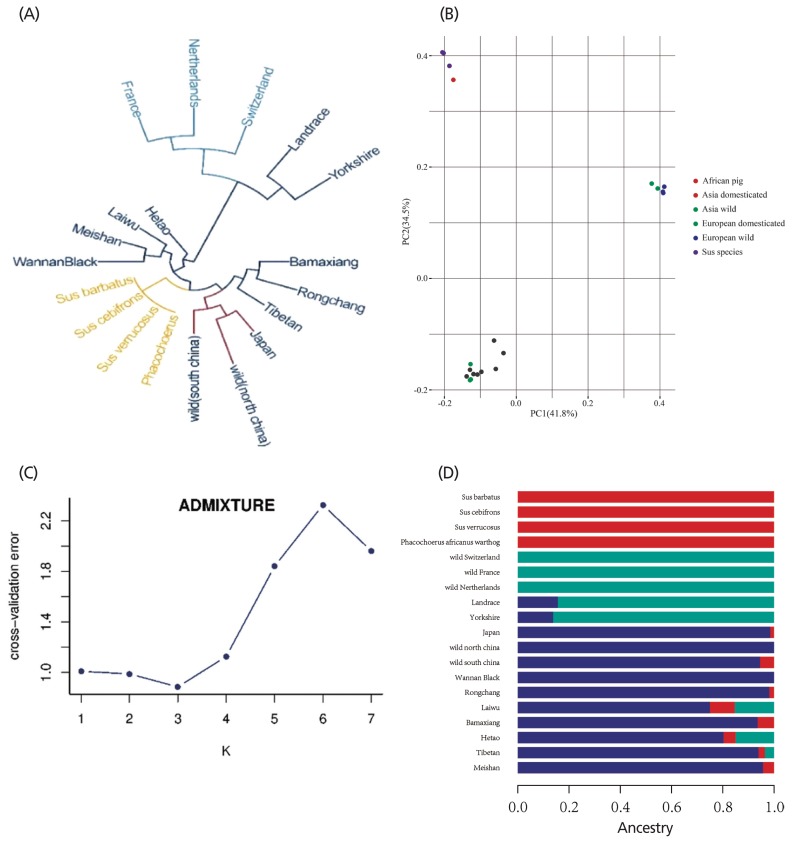
Population genetic structure of pig populations. (A) Neighbor-joining tree constructed from single-nucleotide variants (SNVs) data among 19 subspecies. (B) Principle component analysis plot of pig populations. Different colors represent different subspecies. (C) Cross-validation errors for diverse *k* values. (D) Population structure of study population. The length of different colors represents proportions of ancestry from ancestral populations; breed names are indicated on the left.

After quality control, 903,292 variants were used to construct the neighbor-joining tree and run PCA and admixture analysis. To assess the phylogenetic relationship among the pig breeds in this study, unrooted phylogenetic trees were constructed from the variants after filtering (Figure 3A). The branches of the phylogenetic tree were grouped as expected and were consistent with the results of PCA (Figure 3B), thus revealing strong clustering into three distinct genetic groups comprising Asian wild and domesticated boar, European wild and domesticated pigs, and the other four breeds. To infer population admixture, we chose the lowest cross-validation error value (*k* = 3), which was taken as the most probable number of inferred populations (Figure 3C). Three clusters were observed: *Phacochoerus africanus* and *Sus* species, European wild and domesticated pigs, and Asian wild and domesticated pigs. The results of admixture analysis are shown in Figure 3D with a similar cluster pattern to those found in the PCA plots.

### Selection detected by F_ST_ and π ratio

**Figure 4:**
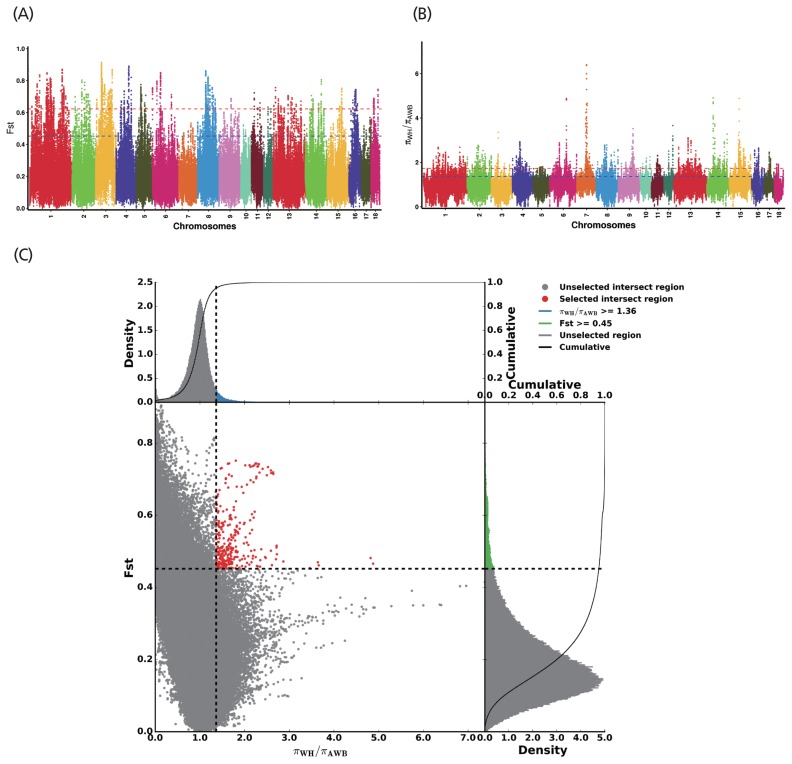
Distribution of F_ST_ values and π ratios calculated in 100 kb windows with 10 kb steps. (A) Distribution of F_ST_ values among autosome chromosomes. The red line represents the 0.01 level, and the blue line represents the 0.05 level. (B) Distribution of π ratios among autosomal chromosomes. (C) Intersection of the two methods used to identify high-quality selection regions. Data points located to the right of vertical dashed line (corresponding to the 5% right tails of the empirical θπ ratio distribution), and above the horizontal dashed line (the 5% right tail of the empirical F_ST_ distribution) were identified as selected regions for Wannan black pig (red points).

**Figure 5:**
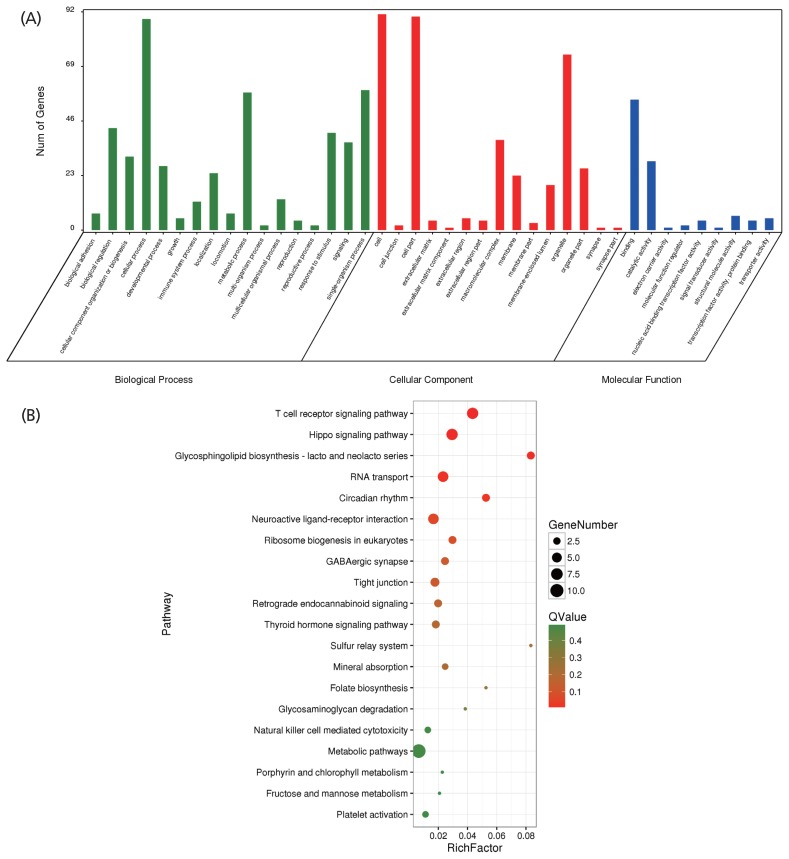
Results of the gene ontology (GO) and Kyoto encyclopedia of genes and genomes (KEGG) analyses for the genes harbored in the selection regions. (A) GO terms of the identified genes. (B) Top 20 enrichment pathways.

After quality control of the SNVs used for selection signature identification, there were 17,067,382 variants. Of these, 16,510,132 were shared with Asian wild boars. The average nucleotide diversity was 0.00213 and 0.00227 for Wannan Black pigs and Asian wild boars, respectively. The genome distribution of the two statistics is shown in Figure 4A and 4B. Twenty-eight selected regions were identified as having extremely high F_ST_ values (1%) and significantly high π ratios (1%) (Supplementary Table S2). Five genes were identified within the regions (Supplementary Table S3); 286 selective regions (threshold, 5%; F_ST_, 0.45; π ratio, 1.36) were identified in Wannan Black pigs (Figure 4C, Supplementary Table S4), which harbored 105 genes (Supplementary Table S5). For further analysis of the genes identified by DAVID, 41 GO terms were identified (Supplementary Table S6). The clusters were related to “reproductive process” “immune system process” “response to stimulus” and “growth” (Figure 5A). For KEGG analysis, 44 pathways were enriched, most of which were related to the immune system, signal transduction, and environmental adaptation, such as “T-cell receptor signaling pathway” “hippo signaling pathway” “Circadian rhythm” and “RNA transport” (Figure 5B, Supplementary Table S7).

## DISCUSSION

Global meat production relies heavily on the capacity and effectiveness of pig breeding. To better understand the genetics underlying their domestication, we performed whole-genome sequencing on 20 Wannan Black pigs and downloaded sequencing data of 28 individuals. To our knowledge, this is the first study to characterize the genetic variation, phylogenetic relationships, population structure, and domestication of the Wannan Black pig. We observed 21 M SNVs and 3 M InDels in the Wannan Black pig genome and a low nucleotide diversity, compared to Asian wild boars.

To reveal the selection signatures of the Wannan Black pig during domestication and breeding, we first selected regions within the top 1% of F_ST_ and π ratios and found five protein coding genes: peptidylprolyl isomerase domain and WD repeat containing 1, which is one of the three classes of peptidylprolyl isomerases found in all eukaryotic and prokaryotic organisms, and viruses, assisting in protein folding [35]; ADAM metallopeptidase with thrombospondin type 1 motif 6, which has been found to be associated with cardiac conduction [36]; tripartite motif containing 23, whose function remains largely unknown; collagen type V alpha 2 chain, which has been shown to be associated with aortic aneurysms and dissections [37]; and centromere protein K, which has been shown to be associated with catastrophic chromosome segregation defects [38]. GO and KEGG analysis of six genes showed significant association with the digestive system and metabolism, which to some extent may indicate their involvement in the crude-feed tolerance of the Wannan Black pig breed.

When we relaxed the threshold from 1% to 5%, a total of 105 genes were identified in 286 selective regions. The genes were significantly enriched in terms of the immune system, environmental adaption, and signal transduction, such as “T-cell receptor signaling pathway” (seven genes), “hippo signaling pathway” (seven genes), “digestive system” (mineral absorption, two genes) “glycosphingolipid biosynthesis—lacto and neolacto series” (three genes), “RNA transport” (six genes)”, “circadian rhythm” (three genes). The T-cell receptor signaling pathway can regulate generic and specialized functions, leading to T-cell proliferation, and cytokine production and differentiation into cells [39]. Hippo signaling is an evolutionarily-conserved signaling pathway that controls organ size in a variety of organisms from flies to humans [40]. The mineral absorption pathway can regulate intestinal calcium transport [41].

The GO analysis identified “reproductive process” (two genes), “immune system process” (12 genes), “growth” (five genes), “response to stimulus” (41 genes), and “molecular function regulator” (two genes). Of the two genes found for this process, Bardet-Biedl syndrome 4 can directly affect the proliferation and differentiation of adipocytes [42]. The immune system process is an organismal system for calibrated responses to potential internal or invasive threats. Of the 12 genes associated with the immune system, zinc finger protein 366 encodes the RNA-binding tristetraprolin, which is needed for CD8^+^ T-cell production of interferon-γ (IFN-γ) *in vivo*. IFN-γ produces cytotoxic T lymphocytes that are essential for host defense against viral infection and cancer [43]. Polymorphisms at the tumor necrosis factor (TNF) superfamily member 15 locus, which encodes the TNF superfamily cytokine commonly known as tumor necrosis factor-like ligand 1A, are associated with susceptibility to inflammatory bowel disease in a range of people [44]. A SH2 domain containing 1A gene mutation in pediatric patients can regulate B-cell lymphoma [45]. The PAF1 homolog, Paf1/RNA polymerase II complex component gene can regulate RNA polymerase II (Pol2) movement through chromatin and the co-transcriptional processing and fate of nascent transcripts [46]. Macrophage stimulating 1 can regulate post-infarction cardiac injury through the JNK-Drp1-mitochondrial fission pathway [47]. Our previous research on MicroRNA-21 and microRNA-214 revealed that they play an important role in the regulation of estrous during porcine reproduction [20]. Analysis of the natural resistance-associated macrophage protein 1 encoding gene in Wannan black pig and Yorkshire identified a single nucleotide polymorphism, which was significantly associated with level of white blood cell % (p = 0.04), monocyte % (p = 0.024), rate of cyotoxin in monocyte % (p = 0.013) and CD4^−^CD8^+^ T lymphocyte (p = 0.023) elucidating the disease-resistance of Wannan black pig compared to Yorkshire [19]. Our previous and whole-genome resequencing results suggest that the genes detailed above played crucial roles in Wannan Black pig domestication by altering the functional regulation of the immune system, environmental adaption, fertility, and the digestive system.

Glycan biosynthesis- and metabolism-related genes have also been under selection pressure in Wannan Black pigs. ST3 beta-galactoside alpha-2,3-sialyltransferase 6 plays a key role in selectin ligand synthesis in humans through the generation of functional sialyl Lewis X. In MRC IX patients, high expression of this gene is associated with lesser overall survival [48].

Growth-related genes were also found to be under strong selection in Wannan Black pigs. Bovine dilated cardiomyopathy (DCM) is an autosomal recessive genetic disorder causing congestive heart failure and subsequent death. Recently, a nonsense mutation, c.343C>T, in the bovine optic atrophy 3 gene reportedly caused DCM in Holstein cattle in Switzerland [49]. A *de novo* mutation in transducin β-like 1 X-linked receptor 1 was found to be associated with autism spectrum disorder and epilepsy by altering Wnt/β-catenin signaling activity [50].

## Figures and Tables

**Table 1:** Summary statistics of whole-genome sequencing

ID	Gender	Raw data (G)	Clean data (G)	Clean reads (M)	Reads mapped (%)	Coverage depth	Coverage at least 1 or 4× (%)
WHJ3	M	21.86	21.75	72,511,844	83.44	8.22	98.59 or 89.30
WHJ23	F	25.38	25.25	84,182,214	85.66	9.54	98.47 or 93.95
WHJ5	M	26.64	26.51	88,379,210	84.84	10.02	98.89 or 94.23
WHJ4	M	26.86	26.61	88,695,743	83.19	10.10	98.40 or 89.83
WHJ6	M	25.25	25.13	83,773,870	84.43	9.50	98.80 or 93.18
WHJ29	F	24.67	24.52	81,746,276	84.97	9.28	98.30 or 91.54
WHJ22	F	24.23	24.10	80,334,486	85.41	9.11	98.52 or 92.99
WHJ21	F	19.93	19.80	66,003,507	84.60	7.50	95.73 or 75.74
WHJ24	F	25.54	25.36	84,541,351	85.09	9.61	98.24 or 90.27
WHJ1	M	21.43	21.32	71,059,903	84.51	8.06	96.33 or 78.76
WHJ18	F	20.21	20.09	66,954,296	84.94	7.60	95.82 or 76.39
WHJ11	M	30.20	30.01	100,031,210	84.17	11.36	98.88 or 94.61
WHJ20	F	26.46	26.34	87,800,323	85.25	9.95	98.54 or 94.59
WHJ19	F	18.87	18.77	62,584,469	85.47	7.10	95.16 or 73.75
WHJ8	M	30.09	29.96	99,860,832	84.39	11.32	98.94 or 95.69
WHJ12	M	26.00	25.82	86,059,643	84.64	9.78	98.30 or 89.29
WHJ16	M	30.27	30.13	100,431,981	84.28	11.38	98.98 or 95.83
WHJ27	F	22.75	22.59	75,304,463	85.47	8.56	97.86 or 86.43
S17	M	26.99	26.89	89,634,691	84.46	10.15	98.93 or 94.44
WHJ28	F	27.89	27.80	92,660,286	85.66	10.49	98.63 or 95.35

**Table 2:** Summary of identified single nucleotide variants and insertions/deletions

Variant type	No. of variants
SNV	21,316,754
Intergenic	12,722,683
Intragenic	8,347,223
Downstream	124,895
Upstream	121,953
Splicing site	1198
5′ UTR	44,897
3′ UTR	198,836
Intron	7,892,061
Coding domain	210,231
Synonymous	144,815
Non-synonymous	64,671
InDel	5,067,206
Intergenic	3,014,421
Intragenic	1,989,666
Downstream	32,210
Upstream	30,909
Splicing site	3840
5′ UTR	10,002
3′ UTR	50,007
Intron	1,912,051
Coding domain	13,766
Frameshift deletion	3,855
Frameshift insertion	6,447
Non-frameshift deletion	1,520
Non-frameshift insertion	1,641

SNVs, single nucleotide variants; InDels, insertions/deletions; UTR, untranslated region.
