# Supplementary material for: Genomic analysis reveals selection signatures of the Wannan Black pig during domestication and breeding
Source: Asian-Australas J Anim Sci. 2019 Aug 23;33(5):712–21. doi: 10.5713/ajas.19.0289 (PMC7206397; doi:10.5713/ajas.19.0289)
Supplement: Supplementary file 1 [file ajas-19-0289-suppl.pdf]

Table S1

Abbreviations	Breed name	Accession number
EWB	European wild boar（ France，Nertherlands， Switizehand）	SAMEA1557387,SAMEA1557401,SAMEA1557403
MS	Meishan	SAMEA3497800
HT	Hetao	SAMN02298115
XIANG	Bamaxiang	SAMN02298127
LR	Landrace	SAMEA1557390
LW	Yorkshire	SAMEA1557383
MIN	Min	SAMN02298121
RC	Rongchang	SAMN02460622
LWU	Laiwu	SAMN02298133
TB	Tibetan	SAMN02298094
AWB	Asia wild boar	SAMEA1557396,SAMEA1557411,SAMEA1557421,SAMN03031180,SAMN03031179,SAMN03031178,SAMN03031177,SAMN03031176,SAMN03031175,SAMN03031174 ,SAMN03031173.SAMN03031172,SAMN03031171
Yucatan	Yucatan	SAMN03031159
Wart	Phacochoerus africanus warthog	SAMEA1557418
Sbar	Sus barbatus	SAMEA1557429
Secb	Sus cebifrons	SAMEA1557409
Sver	S. verrucosus	SAMEA1557384

Table S2

Chr	Start	End	Fst	Pi_WH/Pi_AWB
15	93780001	93880000	0.751	1.803
16	43760001	43860000	0.679	1.824
16	43770001	43870000	0.679	1.935
16	43780001	43880000	0.685	2.053
16	43790001	43890000	0.692	2.199
16	43800001	43900000	0.702	2.260
16	43810001	43910000	0.707	2.265
16	43820001	43920000	0.711	2.498
16	43830001	43930000	0.717	2.596
16	43840001	43940000	0.715	2.644
16	43850001	43950000	0.719	2.638
16	43860001	43960000	0.724	2.588
16	43870001	43970000	0.734	2.524
16	43880001	43980000	0.736	2.302
16	43890001	43990000	0.736	2.182
16	43900001	44000000	0.734	2.133
16	43910001	44010000	0.739	2.111
16	43920001	44020000	0.738	2.015
16	43930001	44030000	0.739	2.216
16	43940001	44040000	0.744	2.245
16	43950001	44050000	0.742	2.320
16	43960001	44060000	0.743	2.266
16	43970001	44070000	0.735	2.289
16	43980001	44080000	0.729	2.403
16	43990001	44090000	0.718	2.333
16	44000001	44100000	0.697	2.133
16	44010001	44110000	0.662	1.972
16	44020001	44120000	0.639	1.807

Table S3

GeneID	Symbol	Description
ENSSSCG0000016945	PPWD1	peptidylprolyl isomerase domain and WD repeat containing 1 [Source:HGNC Symbol;Acc:HGNC:28954]
ENSSSCG0000016943	ADAMTS6	ADAM metallopeptidase with thrombospondin type 1 motif 6 [Source:HGNC Symbol;Acc:HGNC:222]
ENSSSCG0000016948	TRIM23	tripartite motif containing 23 [Source:HGNC Symbol;Acc:HGNC:660]
ENSSSCG0000016035	COL5A2	collagen type V alpha 2 chain [Source:HGNC Symbol;Acc:HGNC:2210]
ENSSSCG0000016946	CENPK	centromere protein K [Source:HGNC Symbol;Acc:HGNC:29479]

Table S4

Chr	Start	End	Fst	Pi_WH/Pi_AWB
1	116850001	116950000	0.658	1.405
1	118570001	118670000	0.454	1.677
1	123040001	123140000	0.536	1.437
1	123050001	123150000	0.505	1.546
1	123060001	123160000	0.482	1.624
1	123070001	123170000	0.454	1.701
1	139950001	140050000	0.465	1.422
1	139960001	140060000	0.496	1.427
1	154190001	154290000	0.456	1.405
1	154210001	154310000	0.465	1.719
1	154220001	154320000	0.480	1.685
1	154230001	154330000	0.497	1.707
1	154240001	154340000	0.470	1.721
1	154250001	154350000	0.487	1.698
1	154260001	154360000	0.489	1.622
1	154300001	154400000	0.459	1.365
1	154440001	154540000	0.512	1.481
1	154450001	154550000	0.512	1.494
1	154460001	154560000	0.494	1.580
1	154470001	154570000	0.465	1.549
1	155710001	155810000	0.554	1.519
1	155720001	155820000	0.530	1.632
1	155730001	155830000	0.522	1.667
1	155740001	155840000	0.504	1.718
1	155750001	155850000	0.521	1.548
1	155760001	155860000	0.556	1.377
1	155960001	156060000	0.633	1.364
1	155970001	156070000	0.571	1.826
1	155980001	156080000	0.484	2.249
1	169700001	169800000	0.465	1.695
1	169710001	169810000	0.466	1.400
1	227700001	227800000	0.474	1.652
1	227710001	227810000	0.491	1.641
1	227720001	227820000	0.483	1.452
1	255240001	255340000	0.453	1.796
1	255250001	255350000	0.488	1.979

1	255260001	255360000	0.478	1.761
1	255270001	255370000	0.470	1.714
1	264710001	264810000	0.478	1.710
1	264720001	264820000	0.478	1.644
1	264730001	264830000	0.459	1.529
2	73240001	73340000	0.455	1.434
2	73250001	73350000	0.454	1.734
2	73270001	73370000	0.511	2.713
2	73280001	73380000	0.516	2.717
2	73290001	73390000	0.524	2.049
2	73300001	73400000	0.521	1.681
2	93540001	93640000	0.458	2.346
2	93550001	93650000	0.458	2.294
2	115140001	115240000	0.552	1.429
2	115150001	115250000	0.570	1.364
2	115160001	115260000	0.573	1.412
2	115170001	115270000	0.593	1.691
2	115180001	115280000	0.614	1.759
2	115190001	115290000	0.622	1.759
2	115200001	115300000	0.603	2.164
2	115210001	115310000	0.610	2.226
2	115220001	115320000	0.568	1.823
3	80210001	80310000	0.511	1.689
3	80220001	80320000	0.499	1.801
3	82340001	82440000	0.478	1.943
3	82350001	82450000	0.461	1.829
3	82360001	82460000	0.454	1.657
3	87960001	88060000	0.478	1.376
3	109480001	109580000	0.453	1.414
3	109490001	109590000	0.465	1.401
3	109500001	109600000	0.456	1.476
4	49940001	50040000	0.461	1.597
4	49950001	50050000	0.482	1.499
4	49960001	50060000	0.507	1.380
4	80480001	80580000	0.520	1.365
4	82730001	82830000	0.458	1.378
5	26220001	26320000	0.460	1.379
6	25230001	25330000	0.460	1.419

6	47970001	48070000	0.489	1.548
6	47980001	48080000	0.494	1.579
6	47990001	48090000	0.475	1.605
6	51960001	52060000	0.461	1.386
6	51970001	52070000	0.489	1.460
6	104810001	104910000	0.468	2.054
6	104820001	104920000	0.499	1.842
6	104830001	104930000	0.502	1.806
6	104840001	104940000	0.485	1.803
6	104850001	104950000	0.475	1.894
6	104860001	104960000	0.480	2.005
6	104870001	104970000	0.486	2.082
6	104880001	104980000	0.501	2.147
6	104890001	104990000	0.503	2.221
6	104900001	105000000	0.518	2.085
6	104910001	105010000	0.506	2.374
6	104920001	105020000	0.472	2.868
6	104930001	105030000	0.454	3.458
6	104940001	105040000	0.466	4.875
6	104950001	105050000	0.482	4.818
6	104960001	105060000	0.470	3.639
7	60500001	60600000	0.460	2.276
7	60510001	60610000	0.476	2.351
7	60520001	60620000	0.484	2.460
7	60530001	60630000	0.482	2.473
7	60540001	60640000	0.478	2.702
8	46480001	46580000	0.468	1.640
8	46490001	46590000	0.463	1.595
8	46910001	47010000	0.586	1.373
8	48700001	48800000	0.465	1.578
8	48710001	48810000	0.480	1.667
8	48720001	48820000	0.473	1.587
8	48730001	48830000	0.470	1.506
8	48740001	48840000	0.465	1.456
8	48750001	48850000	0.458	1.382
8	64720001	64820000	0.468	1.420
8	64730001	64830000	0.460	1.546
8	93950001	94050000	0.463	2.106

8	93990001	94090000	0.507	1.709
8	94000001	94100000	0.578	1.418
8	102650001	102750000	0.460	1.533
8	105640001	105740000	0.464	1.649
9	52180001	52280000	0.474	1.583
9	52190001	52290000	0.480	1.440
9	52200001	52300000	0.498	1.389
9	74690001	74790000	0.490	1.398
9	74700001	74800000	0.501	1.453
9	74710001	74810000	0.501	1.506
9	74720001	74820000	0.507	1.611
9	74730001	74830000	0.492	1.681
9	74740001	74840000	0.484	1.841
9	74750001	74850000	0.484	1.774
9	74760001	74860000	0.467	1.692
9	74770001	74870000	0.454	1.652
9	86460001	86560000	0.462	2.612
9	86470001	86570000	0.492	2.722
9	86480001	86580000	0.502	2.589
9	86490001	86590000	0.488	2.189
9	86500001	86600000	0.480	2.182
9	86510001	86610000	0.470	2.154
9	86610001	86710000	0.458	2.020
9	86620001	86720000	0.459	1.942
9	86630001	86730000	0.455	1.885
9	105300001	105400000	0.526	1.409
9	105310001	105410000	0.547	1.364
9	105330001	105430000	0.574	1.392
12	55140001	55240000	0.461	3.659
13	32220001	32320000	0.536	1.532
13	32230001	32330000	0.579	1.894
13	32240001	32340000	0.593	2.203
13	32250001	32350000	0.586	2.123
13	32260001	32360000	0.579	1.998
13	32270001	32370000	0.576	1.985
13	32280001	32380000	0.563	1.888
13	32290001	32390000	0.549	1.858
13	32300001	32400000	0.544	1.763

13	32310001	32410000	0.507	1.643
13	32320001	32420000	0.478	1.521
13	32330001	32430000	0.472	1.522
13	32340001	32440000	0.465	1.465
13	32350001	32450000	0.453	1.440
13	32360001	32460000	0.464	1.557
13	32370001	32470000	0.455	1.521
13	48470001	48570000	0.508	1.439
13	48480001	48580000	0.500	1.402
13	52130001	52230000	0.454	1.398
13	60460001	60560000	0.469	1.546
13	91410001	91510000	0.453	1.411
13	95480001	95580000	0.460	1.420
13	115450001	115550000	0.464	1.519
13	115460001	115560000	0.508	1.433
13	132800001	132900000	0.498	1.400
13	132810001	132910000	0.544	1.429
13	132820001	132920000	0.561	1.486
13	132830001	132930000	0.566	1.459
13	132840001	132940000	0.574	1.524
13	132850001	132950000	0.583	1.559
13	149120001	149220000	0.498	1.690
13	149130001	149230000	0.537	1.747
13	149140001	149240000	0.555	1.818
13	149150001	149250000	0.557	1.812
13	149160001	149260000	0.560	1.781
13	149170001	149270000	0.531	1.705
13	149180001	149280000	0.509	1.742
13	149190001	149290000	0.517	1.649
13	149200001	149300000	0.545	1.449
13	160190001	160290000	0.509	1.489
13	160200001	160300000	0.505	1.734
13	160210001	160310000	0.513	1.808
13	160220001	160320000	0.550	2.119
13	160230001	160330000	0.559	2.167
13	160240001	160340000	0.530	1.789
13	160250001	160350000	0.511	1.600
13	160260001	160360000	0.502	1.543

13	160270001	160370000	0.493	1.506
13	160280001	160380000	0.492	1.478
13	160290001	160390000	0.488	1.474
13	160300001	160400000	0.477	1.472
14	29620001	29720000	0.463	1.363
14	49280001	49380000	0.458	1.569
14	49290001	49390000	0.475	1.540
14	49300001	49400000	0.455	1.429
14	85630001	85730000	0.601	1.684
14	85640001	85740000	0.598	1.676
14	85650001	85750000	0.583	1.426
14	110410001	110510000	0.471	1.425
14	110420001	110520000	0.469	1.457
14	110430001	110530000	0.463	1.394
15	92590001	92690000	0.474	1.375
15	92600001	92700000	0.571	1.526
15	92780001	92880000	0.535	1.655
15	92790001	92890000	0.574	1.669
15	92800001	92900000	0.579	1.513
15	92810001	92910000	0.607	1.402
15	92820001	92920000	0.630	1.381
15	92840001	92940000	0.652	1.391
15	92850001	92950000	0.630	1.375
15	92860001	92960000	0.612	1.413
15	92870001	92970000	0.568	1.489
15	92880001	92980000	0.498	1.570
15	92890001	92990000	0.465	1.516
15	92900001	93000000	0.467	1.409
15	93760001	93860000	0.729	1.462
15	93770001	93870000	0.743	1.626
15	93780001	93880000	0.751	1.803
15	93790001	93890000	0.744	1.655
15	93800001	93900000	0.731	1.489
15	94310001	94410000	0.651	1.381
15	94320001	94420000	0.637	1.365
15	94330001	94430000	0.625	1.459
15	94340001	94440000	0.616	1.601
15	94350001	94450000	0.597	1.666

15	94360001	94460000	0.572	1.626
15	94370001	94470000	0.546	1.697
15	94380001	94480000	0.507	1.703
15	94390001	94490000	0.503	1.606
15	94400001	94500000	0.473	1.512
16	32420001	32520000	0.578	1.485
16	32430001	32530000	0.589	1.710
16	32440001	32540000	0.594	1.797
16	32450001	32550000	0.590	1.668
16	32460001	32560000	0.568	1.433
16	32470001	32570000	0.569	1.430
16	32480001	32580000	0.548	1.391
16	32490001	32590000	0.535	1.364
16	32500001	32600000	0.534	1.374
16	32510001	32610000	0.479	1.367
16	36210001	36310000	0.703	1.581
16	37610001	37710000	0.497	1.510
16	37620001	37720000	0.510	1.601
16	37630001	37730000	0.504	1.637
16	37640001	37740000	0.514	1.514
16	37650001	37750000	0.526	1.419
16	37660001	37760000	0.509	1.413
16	42680001	42780000	0.454	1.589
16	42690001	42790000	0.456	1.517
16	43700001	43800000	0.670	1.439
16	43710001	43810000	0.709	1.713
16	43720001	43820000	0.713	1.652
16	43730001	43830000	0.701	1.580
16	43740001	43840000	0.689	1.572
16	43750001	43850000	0.677	1.634
16	43760001	43860000	0.679	1.824
16	43770001	43870000	0.679	1.935
16	43780001	43880000	0.685	2.053
16	43790001	43890000	0.692	2.199
16	43800001	43900000	0.702	2.260
16	43810001	43910000	0.707	2.265
16	43820001	43920000	0.711	2.498
16	43830001	43930000	0.717	2.596

16	43840001	43940000	0.715	2.644
16	43850001	43950000	0.719	2.638
16	43860001	43960000	0.724	2.588
16	43870001	43970000	0.734	2.524
16	43880001	43980000	0.736	2.302
16	43890001	43990000	0.736	2.182
16	43900001	44000000	0.734	2.133
16	43910001	44010000	0.739	2.111
16	43920001	44020000	0.738	2.015
16	43930001	44030000	0.739	2.216
16	43940001	44040000	0.744	2.245
16	43950001	44050000	0.742	2.320
16	43960001	44060000	0.743	2.266
16	43970001	44070000	0.735	2.289
16	43980001	44080000	0.729	2.403
16	43990001	44090000	0.718	2.333
16	44000001	44100000	0.697	2.133
16	44010001	44110000	0.662	1.972
16	44020001	44120000	0.639	1.807
16	44030001	44130000	0.611	1.522
16	49010001	49110000	0.619	1.370
16	49060001	49160000	0.630	1.379

Table S5

GeneID	Symbol	Description
ENSSSCG00000022828	PNN	pinin, desmosome associated protein [Source:NCBI gene;Acc:100526149]
ENSSSCG00000011978	CPOX	coproporphyrinogen oxidase [Source:NCBI gene;Acc:100511474]
ENSSSCG00000006301	TIPRL	TOR signaling pathway regulator [Source:HGNC Symbol;Acc:HGNC:30231]
ENSSSCG00000028056	ZFP36	ZFP36 ring finger protein [Source:NCBI gene;Acc:100316849]
ENSSSCG00000001924	BBS4	Bardet-Biedl syndrome 4 [Source:HGNC Symbol;Acc:HGNC:969]
ENSSSCG00000035584	GABRG3	gamma-aminobutyric acid type A receptor gamma3 subunit [Source:HGNC Symbol;Acc:HGNC:4088]
ENSSSCG00000040981	GMFG	glia maturation factor gamma [Source:HGNC Symbol;Acc:HGNC:4374]
ENSSSCG00000010706	SAMD4B	sterile alpha motif domain containing 4B [Source:NCBI gene;Acc:100516158]
ENSSSCG00000034087	TNFSF15	TNF superfamily member 15 [Source:NCBI gene;Acc:100624969]
ENSSSCG00000012643	SH2D1A	SH2 domain containing 1A [Source:NCBI gene;Acc:780420]
ENSSSCG00000038229	EML2	echinoderm microtubule associated protein like 2 [Source:HGNC Symbol;Acc:HGNC:18035]
ENSSSCG00000001925	ADPGK	ADP dependent glucokinase [Source:HGNC Symbol;Acc:HGNC:25250]
ENSSSCG00000010351	CCSER2	coiled-coil serine rich protein 2 [Source:HGNC Symbol;Acc:HGNC:29197]
ENSSSCG00000011389	AMIGO3	adhesion molecule with Ig like domain 3 [Source:HGNC Symbol;Acc:HGNC:24075]
ENSSSCG00000016042	ANKAR	PREDICTED: LOW QUALITY PROTEIN: ankyrin and armadillo repeat-containing protein-like [Sus scrofa]
ENSSSCG00000027293	TNPO1	transportin 1 [Source:HGNC Symbol;Acc:HGNC:6401]
ENSSSCG00000016035	COL5A2	collagen type V alpha 2 chain [Source:HGNC Symbol;Acc:HGNC:2210]
ENSSSCG00000005268	RORB	RAR related orphan receptor B [Source:HGNC Symbol;Acc:HGNC:10259]
ENSSSCG00000011391	CDHR4	cadherin related family member 4 [Source:HGNC Symbol;Acc:HGNC:34527]
ENSSSCG00000037184	GPR4	G protein-coupled receptor 4 [Source:HGNC Symbol;Acc:HGNC:4497]

ENSSSCG00 000032162	P2RY13	purinergic receptor P2Y13 [Source:HGNC Symbol;Acc:HGNC:4537]
ENSSSCG00 000016976	ZNF366	zinc finger protein 366 [Source:HGNC Symbol;Acc:HGNC:18316]
ENSSSCG00 000016946	CENPK	centromere protein K [Source:HGNC Symbol;Acc:HGNC:29479]
ENSSSCG00 000027992	MST1R	macrophage stimulating 1 receptor [Source:HGNC Symbol;Acc:HGNC:7381]
ENSSSCG00 000022536	SLC37A 2	solute carrier family 37 member 2 [Source:HGNC Symbol;Acc:HGNC:20644]
ENSSSCG00 000004885	CDH19	cadherin 19 [Source:HGNC Symbol;Acc:HGNC:1758]
ENSSSCG00 000028228	XPO1	exportin 1 [Source:HGNC Symbol;Acc:HGNC:12825]
ENSSSCG00 000029492	TMEM21 8	transmembrane protein 218 [Source:HGNC Symbol;Acc:HGNC:27344]
ENSSSCG00 000027128	LONP1	lon peptidase 1, mitochondrial [Source:NCBI gene;Acc:100511917]
ENSSSCG00 000009779	MPHOSP H9	M-phase phosphoprotein 9 [Source:HGNC Symbol;Acc:HGNC:7215]
ENSSSCG00 000022442	PAF1	PAF1 homolog, Paf1/RNA polymerase II complex component [Source:HGNC Symbol;Acc:HGNC:25459]
ENSSSCG00 000011386	MST1	macrophage stimulating 1 [Source:HGNC Symbol;Acc:HGNC:7380]
ENSSSCG00 000011712	P2RY14	purinergic receptor P2Y14 [Source:HGNC Symbol;Acc:HGNC:16442]
ENSSSCG00 000009776	SBNO1	strawberry notch homolog 1 [Source:HGNC Symbol;Acc:HGNC:22973]
ENSSSCG00 000006280	GORAB	golgin, RAB6 interacting [Source:HGNC Symbol;Acc:HGNC:25676]
ENSSSCG00 000031503	PRRX1	paired related homeobox 1 [Source:HGNC Symbol;Acc:HGNC:9142]
ENSSSCG00 000016043	OSGEPL 1	O-sialoglycoprotein endopeptidase like 1 [Source:HGNC Symbol;Acc:HGNC:23075]
ENSSSCG00 000016033	GULP1	GULP, engulfment adaptor PTB domain containing 1 [Source:HGNC Symbol;Acc:HGNC:18649]
ENSSSCG00 000015206	CCDC15	coiled-coil domain containing 15 [Source:HGNC Symbol;Acc:HGNC:25798]
ENSSSCG00 000011393	UBA7	ubiquitin like modifier activating enzyme 7 [Source:HGNC Symbol;Acc:HGNC:12471]
ENSSSCG00 000016887	ITGA2	integrin subunit alpha 2 [Source:HGNC Symbol;Acc:HGNC:6137]
ENSSSCG00 000016888	MOCS2	molybdenum cofactor synthesis 2 [Source:HGNC Symbol;Acc:HGNC:7193]

ENSSSCG00000018005	MYH8	myosin heavy chain 8 [Source:HGNC Symbol;Acc:HGNC:7578]
ENSSSCG00000030484	AHR	aryl hydrocarbon receptor [Source:NCBI gene;Acc:396654]
ENSSSCG00000037100	OPA3	OPA3, outer mitochondrial membrane lipid metabolism regulator [Source:HGNC Symbol;Acc:HGNC:8142]
ENSSSCG00000016943	ADAMTS6	ADAM metallopeptidase with thrombospondin type 1 motif 6 [Source:HGNC Symbol;Acc:HGNC:222]
ENSSSCG00000011713	P2RY12	purinergic receptor P2Y12 [Source:HGNC Symbol;Acc:HGNC:18124]
ENSSSCG00000038367	FBXO46	PREDICTED: LOW QUALITY PROTEIN: F-box only protein 46 [Balaenoptera acutorostrata scammoni]
ENSSSCG00000013524	C19orf70	chromosome 19 open reading frame 70 [Source:HGNC Symbol;Acc:HGNC:33702]
ENSSSCG00000011980	CLDND1	claudin domain containing 1 [Source:HGNC Symbol;Acc:HGNC:1322]
ENSSSCG00000031617	SFT2D2	SFT2 domain containing 2 [Source:NCBI gene;Acc:100157189]
ENSSSCG00000013527	CATSPERD	cation channel sperm associated auxiliary subunit delta [Source:HGNC Symbol;Acc:HGNC:28598]
ENSSSCG00000016045	PMS1	PMS1 homolog 1, mismatch repair system component [Source:HGNC Symbol;Acc:HGNC:9121]
ENSSSCG00000005585	DENND1A	DENN domain containing 1A [Source:HGNC Symbol;Acc:HGNC:29324]
ENSSSCG00000011714	MED12L	mediator complex subunit 12 like [Source:HGNC Symbol;Acc:HGNC:16050]
ENSSSCG00000030546	HSD11B1L	hydroxysteroid 11-beta dehydrogenase 1 like [Source:HGNC Symbol;Acc:HGNC:30419]
ENSSSCG00000026718	PLCH1	phospholipase C eta 1 [Source:HGNC Symbol;Acc:HGNC:29185]
ENSSSCG00000004986	TRAPPC6B	trafficking protein particle complex 6B [Source:HGNC Symbol;Acc:HGNC:23066]
ENSSSCG00000031796	RAB3C	RAB3C, member RAS oncogene family [Source:NCBI gene;Acc:100523703]
ENSSSCG00000004985	GEMIN2	gem nuclear organelle associated protein 2 [Source:HGNC Symbol;Acc:HGNC:10884]
ENSSSCG00000027041	-	ferritin, heavy subunit-like [Source:NCBI gene;Acc:102162930]
ENSSSCG00000010053	SPECC1L	sperm antigen with calponin homology and coiled-coil domains 1 like [Source:NCBI gene;Acc:100519740]
ENSSSCG00000032819	QPCTL	glutaminyl-peptide cyclotransferase like [Source:HGNC Symbol;Acc:HGNC:25952]
ENSSSCG00000029441	MYH2	myosin, heavy chain 2, skeletal muscle, adult [Source:NCBI gene;Acc:397256]

ENSSSCG00000014196	TMEM232	PREDICTED: transmembrane protein 232
ENSSSCG00000032407	LRFN1	leucine rich repeat and fibronectin type III domain containing 1 [Source:HGNC Symbol;Acc:HGNC:29290]
ENSSSCG00000015428	PUS7	pseudouridylate synthase 7 [Source:HGNC Symbol;Acc:HGNC:26033]
ENSSSCG00000016948	TRIM23	tripartite motif containing 23 [Source:HGNC Symbol;Acc:HGNC:660]
ENSSSCG00000021053	GIPR	gastric inhibitory polypeptide receptor [Source:HGNC Symbol;Acc:HGNC:4271]
ENSSSCG00000025416	CAMKV	CaM kinase like vesicle associated [Source:HGNC Symbol;Acc:HGNC:28788]
ENSSSCG00000009098	QRFPR	pyroglutamylated RFamide peptide receptor [Source:HGNC Symbol;Acc:HGNC:15565]
ENSSSCG00000016945	PPWD1	peptidylprolyl isomerase domain and WD repeat containing 1 [Source:HGNC Symbol;Acc:HGNC:28954]
ENSSSCG00000030048	PLEKHG2	pleckstrin homology and RhoGEF domain containing G2 [Source:HGNC Symbol;Acc:HGNC:29515]
ENSSSCG00000011394	RBM6	RNA binding motif protein 6 [Source:HGNC Symbol;Acc:HGNC:9903]
ENSSSCG00000004658	FBN1	fibrillin 1 [Source:NCBI gene;Acc:414836]
ENSSSCG00000011833	DLG1	discs large MAGUK scaffold protein 1 [Source:HGNC Symbol;Acc:HGNC:2900]
ENSSSCG00000012644	TENM1	teneurin transmembrane protein 1 [Source:HGNC Symbol;Acc:HGNC:8117]
ENSSSCG00000034493	ST3GAL6	ST3 beta-galactoside alpha-2,3-sialyltransferase 6 [Source:HGNC Symbol;Acc:HGNC:18080]
ENSSSCG00000004983	MIA2	MIA SH3 domain ER export factor 2 [Source:NCBI gene;Acc:100155295]
ENSSSCG00000010535	HPSE2	heparanase 2 (inactive) [Source:HGNC Symbol;Acc:HGNC:18374]
ENSSSCG00000011387	RNF123	ring finger protein 123 [Source:HGNC Symbol;Acc:HGNC:21148]
ENSSSCG00000009775	CDK2AP1	cyclin-dependent kinase 2-associated protein 1 [Sus scrofa]
ENSSSCG00000029857	EIF2S3	eukaryotic translation initiation factor 2 subunit gamma [Source:NCBI gene;Acc:100525970]
ENSSSCG00000010052	BCR	BCR, RhoGEF and GTPase activating protein [Source:HGNC Symbol;Acc:HGNC:1014]
ENSSSCG00000013523	SAFB2	scaffold attachment factor B2 [Source:HGNC Symbol;Acc:HGNC:21605]
ENSSSCG00000033410	INKA1	inka box actin regulator 1 [Source:HGNC Symbol;Acc:HGNC:32480]

ENSSSCG00000025791	MON1A	MON1 homolog A, secretory trafficking associated [Source:NCBI gene;Acc:100626383]
ENSSSCG00000026977	TRAIP	TRAF interacting protein [Source:HGNC Symbol;Acc:HGNC:30764]
ENSSSCG00000032365	GPR87	G protein-coupled receptor 87 [Source:HGNC Symbol;Acc:HGNC:4538]
ENSSSCG00000011979	GPR15	G protein-coupled receptor 15 [Source:HGNC Symbol;Acc:HGNC:4469]
ENSSSCG00000020817	RPS16	ribosomal protein S16 [Source:NCBI gene;Acc:414397]
ENSSSCG00000015329	PPP1R9A	protein phosphatase 1 regulatory subunit 9A [Source:HGNC Symbol;Acc:HGNC:14946]
ENSSSCG00000033500	SNRPD2	small nuclear ribonucleoprotein D2 polypeptide [Source:NCBI gene;Acc:100626720]
ENSSSCG00000009774	C12orf65	chromosome 12 open reading frame 65 [Source:HGNC Symbol;Acc:HGNC:26784]
ENSSSCG00000013522	SAFB	scaffold attachment factor B [Source:HGNC Symbol;Acc:HGNC:10520]
ENSSSCG00000021322	ZFX	zinc finger protein X-linked [Source:NCBI gene;Acc:397294]
ENSSSCG00000011390	IP6K1	inositol hexakisphosphate kinase 1 [Source:HGNC Symbol;Acc:HGNC:18360]
ENSSSCG00000015430	RINT1	RAD50 interactor 1 [Source:HGNC Symbol;Acc:HGNC:21876]
ENSSSCG00000005269	TRPM6	transient receptor potential cation channel subfamily M member 6 [Source:HGNC Symbol;Acc:HGNC:17995]
ENSSSCG00000011500	KBTBD8	kelch repeat and BTB domain containing 8 [Source:HGNC Symbol;Acc:HGNC:30691]
ENSSSCG00000018003	MYH4	myosin heavy chain 4 [Source:HGNC Symbol;Acc:HGNC:7574]
ENSSSCG00000011760	TBL1XR1	transducin beta like 1 X-linked receptor 1 [Source:HGNC Symbol;Acc:HGNC:29529]
ENSSSCG00000016941	RNF180	ring finger protein 180 [Source:HGNC Symbol;Acc:HGNC:27752]
ENSSSCG00000016044	ORMDL1	ORMDL sphingolipid biosynthesis regulator 1 [Source:NCBI gene;Acc:100155491]
ENSSSCG00000016922	GPBP1	GC-rich promoter binding protein 1 [Source:HGNC Symbol;Acc:HGNC:29520]

Table S6

Ontology	Class
Biological Process	reproductive process
Biological Process	cellular component organization or biogenesis
Biological Process	cellular process
Biological Process	signaling
Biological Process	biological regulation
Biological Process	immune system process
Biological Process	biological adhesion
Biological Process	metabolic process
Biological Process	growth
Biological Process	response to stimulus
Biological Process	developmental process
Biological Process	multi-organism process
Biological Process	locomotion
Biological Process	localization
Biological Process	reproduction
Biological Process	multicellular organismal process
Biological Process	single-organism process
Molecular Function	electron carrier activity
Molecular Function	signal transducer activity
Molecular Function	binding
Molecular Function	transcription factor activity, protein binding
Molecular Function	catalytic activity
Molecular Function	structural molecule activity
Molecular Function	transporter activity
Molecular Function	nucleic acid binding transcription factor activity
Molecular Function	molecular function regulator
Cellular Component	organelle
Cellular Component	macromolecular complex
Cellular Component	extracellular matrix component
Cellular Component	cell junction
Cellular Component	membrane part
Cellular Component	extracellular matrix
Cellular Component	synapse part
Cellular Component	synapse
Cellular Component	cell part
Cellular Component	organelle part

Cellular Component	cell
Cellular Component	membrane-enclosed lumen
Cellular Component	membrane
Cellular Component	extracellular region part
Cellular Component	extracellular region

Table S7

KEGG_A_class	KEGG_B_class	Pathway	Pvalue	Qvalue	Genes
Organismal Systems	Immune system	T cell receptor signaling pathway	0.000	0.001	ENSSSCG00000011833;ENSSSCG0000001
Environmental Information Processing	Signal transduction	Hippo signaling pathway	0.000	0.003	ENSSSCG00000011833;ENSSSCG0000001
Metabolism	Glycan biosynthesis and metabolism	Glycosphingolipid biosynthesis - lacto and neolacto series	0.001	0.011	ENSSSCG00000034493;ENSSSCG0000003
Genetic Information Processing	Translation	RNA transport	0.002	0.018	ENSSSCG00000004985;ENSSSCG0000002
Organismal Systems	Environmental adaptation	Circadian rhythm	0.003	0.025	ENSSSCG00000005268;ENSSSCG0000000
Environmental Information Processing	Signaling molecules and interaction	Neuroactive ligand-receptor interaction	0.008	0.061	ENSSSCG00000011712;ENSSSCG0000002
Genetic Information Processing	Translation	Ribosome biogenesis in eukaryotes	0.014	0.086	ENSSSCG00000028228;ENSSSCG0000002
Organismal Systems	Nervous system	GABAergic synapse	0.023	0.125	ENSSSCG00000035584;ENSSSCG0000003
Cellular Processes	Cellular commiunity	Tight junction	0.026	0.125	ENSSSCG00000018003;ENSSSCG0000001
Organismal Systems	Nervous system	Retrograde endocannabinoid signaling	0.039	0.172	ENSSSCG00000035584;ENSSSCG0000003
Organismal Systems	Endocrine system	Thyroid hormone signaling pathway	0.048	0.192	ENSSSCG00000011714;ENSSSCG0000001
Genetic Information Processing	Folding, sorting and degradation	Sulfur relay system	0.058	0.209	ENSSSCG00000016888

Organismal Systems	Digestive system	Mineral absorption	0.062	0.209	ENSSSCG00000005269;ENSSSCG0000002
Metabolism	Metabolism of cofactors and vitamins	Folate biosynthesis	0.091	0.286	ENSSSCG00000016888
Metabolism	Glycan biosynthesis and metabolism	Glycosaminoglycan degradation	0.122	0.359	ENSSSCG00000010535
Organismal Systems	Immune system	Natural killer cell mediated cytotoxicity	0.183	0.485	ENSSSCG00000012643;ENSSSCG0000001
Metabolism	Global and Overview	Metabolic pathways	0.197	0.485	ENSSSCG00000001925;ENSSSCG0000001
Metabolism	Metabolism of cofactors and vitamins	Porphyrin and chlorophyll metabolism	0.198	0.485	ENSSSCG00000011978
Metabolism	Carbohydrate metabolism	Fructose and mannose metabolism	0.214	0.491	ENSSSCG00000011389
Organismal Systems	Immune system	Platelet activation	0.223	0.491	ENSSSCG00000011713;ENSSSCG0000001
Metabolism	Global and Overview	Biosynthesis of secondary metabolites	0.243	0.509	ENSSSCG00000001925;ENSSSCG0000001
Metabolism	Carbohydrate metabolism	Amino sugar and nucleotide sugar metabolism	0.275	0.534	ENSSSCG00000011389
Genetic Information Processing	Transcription	Basal transcription factors	0.279	0.534	ENSSSCG00000030745
Genetic Information Processing	Transcription	Spliceosome	0.300	0.550	ENSSSCG00000033500;ENSSSCG0000003
Metabolism	Carbohydrate metabolism	Glycolysis / Gluconeogenesis	0.365	0.617	ENSSSCG00000001925
Metabolism	Carbohydrate metabolism	Inositol phosphate metabolism	0.365	0.617	ENSSSCG00000026718
Organismal Systems	Immune system	Hematopoietic cell lineage	0.381	0.621	ENSSSCG00000016887
Organismal Systems	Digestive system	Protein digestion and absorption	0.441	0.671	ENSSSCG00000016035
Metabolism	Global and Overview	Biosynthesis of antibiotics	0.444	0.671	ENSSSCG00000001925;ENSSSCG0000003

Environmental Information Processing	Signaling molecules and interaction	ECM-receptor interaction	0.457	0.671	ENSSSCG00000016887
Environmental Information Processing	Signal transduction	Phosphatidylinositol signaling system	0.518	0.713	ENSSSCG00000011390
Metabolism	Global and Overview	Carbon metabolism	0.537	0.713	ENSSSCG00000001925
Genetic Information Processing	Translation	Ribosome	0.546	0.713	ENSSSCG00000015429;ENSSSCG0000002[illegible]
Genetic Information Processing	Translation	mRNA surveillance pathway	0.551	0.713	ENSSSCG00000022828
Cellular Processes	Transport and catabolism	Phagosome	0.632	0.795	ENSSSCG00000016887
Environmental Information Processing	Signal transduction	Wnt signaling pathway	0.660	0.806	ENSSSCG00000011760
Metabolism	Energy metabolism	Oxidative phosphorylation	0.687	0.808	ENSSSCG00000040930
Genetic Information Processing	Folding, sorting and degradation	Ubiquitin mediated proteolysis	0.698	0.808	ENSSSCG00000011393
Metabolism	Nucleotide metabolism	Purine metabolism	0.730	0.821	ENSSSCG00000037817
Environmental Information Processing	Signaling molecules and interaction	Cytokine-cytokine receptor interaction	0.764	0.821	ENSSSCG00000034087
Environmental Information Processing	Signal transduction	cAMP signaling pathway	0.790	0.821	ENSSSCG00000021053
Cellular Processes	Cellular commiunity	Focal adhesion	0.792	0.821	ENSSSCG00000016887
Cellular Processes	Cell motility	Regulation of actin cytoskeleton	0.803	0.821	ENSSSCG00000016887
Environmental Information Processing	Signal transduction	PI3K-Akt signaling pathway	0.912	0.912	ENSSSCG00000016887
